# Protocol for SUMO fusion–based functional rescue of SUMOylation-defective protein in *Drosophila*

**DOI:** 10.1016/j.xpro.2026.104569

**Published:** 2026-05-13

**Authors:** Rin Imai, Yaning Wu, Riku Kawamura, Kensaku Murano

**Affiliations:** 1 Department of Molecular Biology, Keio University School of Medicine, Tokyo, Japan

**Keywords:** Cell-based Assays, Molecular Biology, Gene Expression

## Abstract

Here, we present a protocol for the functional rescue of small ubiquitin-like modifier (SUMO)ylation-defective proteins using a SUMO fusion-based gain-of-function approach. Using Panx involved in the Piwi-piRNA-mediated genome defense in *Drosophila*, we describe the restoration of transcriptional silencing by stable fusion of a non-cleavable SUMO variant. We then detail procedures for the quantitative evaluation of transcriptional repression. This approach enables causal analysis of SUMO-dependent protein function beyond conventional loss-of-function strategies.

For complete details of this protocol, please refer to Wu et al.^1^

## Highlights

• Steps for assessing SUMOylation-dependent protein function using an in-frame SUMO fusion
• Procedures for stable SUMO attachment using a non-cleavable SUMO variant (GG→AA)
• Instructions for quantitative evaluation of transcriptional repression
• Guidance on functional rescue analysis of SUMOylation-defective proteins in cells

## Abstract (editor highlights)

Publisher’s note: Undertaking any experimental protocol requires adherence to local institutional guidelines for laboratory safety and ethics.

## Graphical abstract

**Figure:**
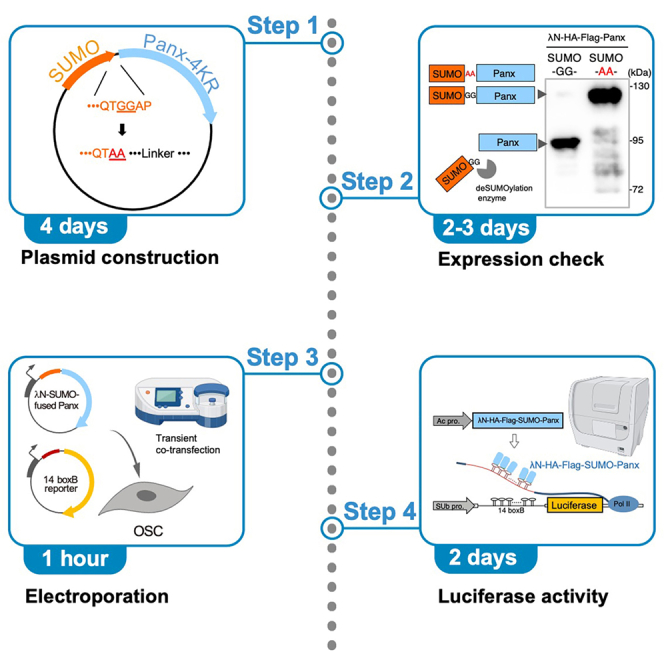

## Teaser

Here, we present a protocol for the functional rescue of small ubiquitin-like modifier (SUMO)ylation-defective proteins using a SUMO fusion-based gain-of-function approach. Using the Piwi-piRNA pathway effector Panx in *Drosophila* ovarian somatic cells, we describe the restoration of transcriptional silencing by stable fusion of a non-cleavable SUMO variant. We then detail procedures for the quantitative evaluation of transcriptional repression. This approach enables causal analysis of SUMO-dependent protein function beyond conventional loss-of-function strategies.

## Before you begin

Small ubiquitin-like modifier (SUMO) modification is a conserved and reversible post-translational modification that regulates protein activity, cellular localization, and protein–protein interactions.^2^^,^^3^ In *Drosophila*, SUMO is encoded by the *Smt3* gene and synthesized as an inactive precursor that is processed to expose a conserved C-terminal diglycine (GG) motif, which is essential for covalent conjugation to substrate proteins.^4^

Protein function is commonly investigated using loss-of-function and gain-of-function approaches. In studies of post-translational modifications, loss-of-function strategies often involve substitution of modification-target residues with non-modifiable amino acids. For SUMOylation, lysine-to-arginine (K→R) substitutions are widely used to prevent SUMO conjugation.^5^ However, because lysine residues are subject to multiple post-translational modifications and may also contribute to protein structure, K→R substitutions can alter protein function independently of SUMOylation.^6^^,^^7^ Therefore, loss-of-function analyses alone are insufficient to define the specific contribution of SUMO attachment. Gain-of-function approaches are therefore required to directly assess the role of this modification. Whereas phosphorylation can be mimicked by substituting serine or threonine residues with acidic amino acids,^8^ SUMOylation is considerably more challenging, because it entails covalent attachment of the SUMO moiety and causes structural and functional effects that cannot be readily recapitulated by simple amino acid substitutions.

**Figure 1:**
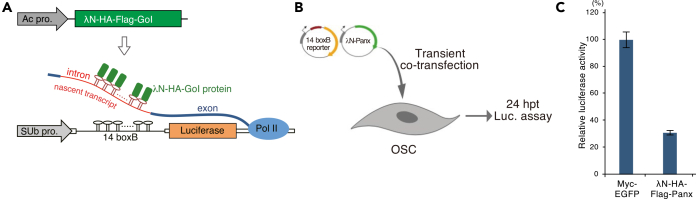
Enforced tethering of λN-Panx shows silencing of reporter gene expression (A) Schematic of the λN–boxB tethering system in OSCs. The luciferase reporter plasmid contains 14 copies of boxB sites inserted into the intronic region of the *Drosophila simulans ubiquitin* gene promoter(SUb). λN fusion proteins are recruited to the nascent luciferase RNA *via* the boxB sites. GoI indicates the gene of interest. (B) Experimental design of the λN–boxB tethering assay. OSCs are co-transfected with the reporter plasmid and an expression plasmid encoding λN-tagged GoI (e.g., Panx). Cells are harvested 24 h post-transfection and subjected to a luciferase assay. (C) As an example, enforced tethering of λN-Panx represses luciferase activity to less than 30% relative to the Myc-EGFP control. Error bars indicate SD (n=3).

**Figure 2:**
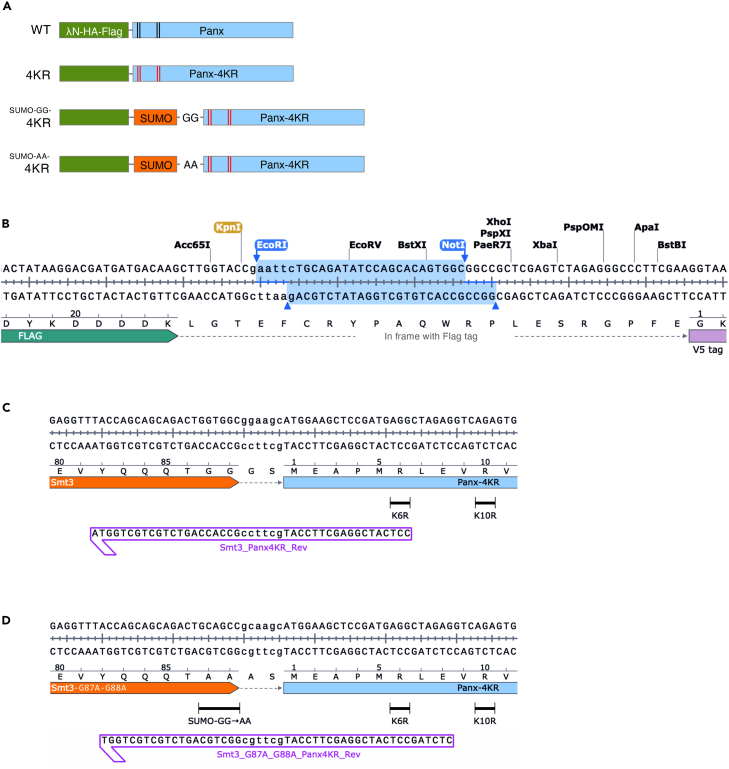
Construction of SUMO-fused Panx-4KR plasmids (A) Schematics of λN-fused Panx variants used in this study. Black and red lines indicate the four N-terminal lysine residues in wild-type Panx and the corresponding K→R substitutions in the 4KR mutant, respectively. (B) Schematic of the λN–HA–Flag expression plasmid. The GoI is inserted between the EcoRI and NotI restriction sites. This design retains a KpnI site immediately upstream of the insert, enabling N-terminal fusion of SUMO. (C) Schematic representation of the plasmid design showing in-frame fusion of SUMO (Smt3) to the N terminal of Panx-4KR. (D) The C-terminal diglycine motif of SUMO is mutated to alanine residues (GG→AA) to generate a non-cleavable SUMO-fused Panx-4KR.

Here, we focus on Panx, a SUMOylated effector of the Piwi–piRNA pathway that mediates transcriptional silencing of transposable elements (TEs) in *Drosophila* ovarian somatic cells (OSCs).^1^^,^^9^^,^^10^ To elucidate the mechanism by which Panx mediates transcriptional silencing of TEs, we employed the λN-boxB tethering system coupled to a luciferase reporter (Figure 1A). λN-fused Panx binds nascent reporter transcripts, leading to repression of reporter gene transcription (Figures 1B and 1C). Substitution of four SUMOylation-targeted lysine residues in the N-terminus of Panx with arginine (the Panx 4KR mutant) results in a loss-of-function phenotype characterized by defective transcriptional repression (Figure 2A). In contrast, genetic fusion of SUMO to the N terminus of the Panx 4KR mutant restores repression activity, even in SUMOylation-deficient backgrounds. This protocol describes the design, expression, and functional evaluation of SUMO-fusion proteins, enabling a gain-of-function analysis to directly assess the role of SUMOylation in protein regulation.

### Innovation

Functional analyses of SUMOylation have traditionally relied on lysine-to-arginine (K→R) mutagenesis to generate loss-of-function mutants. However, loss-of-function approaches alone are insufficient to conclusively demonstrate the functional contribution of SUMOylation. In contrast to phosphorylation, for which gain-of-function effects can often be mimicked using phosphomimetic substitutions, comparable gain-of-function strategies are difficult to implement for SUMOylation. To overcome this limitation, the concept of fusing the SUMO protein in frame to the open reading frame (ORF) of a target protein has been proposed.^11^ Consistent with this idea, fusion of SUMO to the N terminus of the Panx KR mutant partially restored its transcriptional repression activity. However, despite being fused in frame, the SUMO moiety attached to Panx was susceptible to cleavage by SUMO-specific proteases, resulting in its removal from the fusion protein.

To address this issue, this protocol provides a design workflow that incorporates a non-cleavable SUMO variant^12^ in which the C-terminal diglycine motif is mutated to alanine residues (Figure 2A). This strategy ensures stable, protease-resistant SUMO-fusion proteins and enables a practical and broadly applicable gain-of-function approach to interrogate SUMO-dependent mechanisms.

## Key resources table

**Table:** 

REAGENT or RESOURCE	SOURCE	IDENTIFIER
**Antibodies**		

Mouse Monoclonal anti-Panx (10D8) 1:5 dilution	Murano et al.^13^	N/A
Mouse Monoclonal anti-Tubulin (E7) 1:5 dilution	DSHB (Hybridoma Bank)	Cat#: E7 RRID: AB_2315513
Anti-mouse 2^nd^ antibody (HRP-conjugated) 1:5000 dilution	MP Biomedicals	Cat#: 55558

**Bacterial and virus strains**		

*E.coli* Mach1	Thermo Fisher	Cat#: C862003

**Chemicals, peptides, and recombinant proteins**		

KpnI-HF	NEB	Cat#: R3142L
PrimeSTAR Max DNA Polymerase Ver.2	TaKaRa	Cat#: RO47
NEBuilder HiFi DNA Assembly Master Mix	NEB	Cat#: E2621
Ampicillin	Wako	Cat#: 014-23302
UltraPure 0.5 M EDTA, pH 8.0	Thermo Fisher	Cat#: 15575020
ECL Prime	Cytiva	Cat#: RPN 2236
Insulin	Sigma	Cat#: I6634-100MG
Glutathione	Sigma	Cat#: G6013-10G
Shields and Sang M3 Insect Medium w/L-Glutamine (Powder)	US Biological	Cat#: S1013
Penicillin/Streptomycin	Gibco	Cat#: 15140122
D-Mannitol	Sigma	Cat#: M4125-1KG
Sodium chloride	Wako	Cat#: 191-01665
Magnesium chloride	Wako	Cat#: 135-00165
Disodium Hydrogenphosphate	Wako	Cat#: 040-30051
Phosphoric Acid	Wako	Cat#: 167-02166
Sodium dodecyl sulfate (SDS)	Wako	Cat#: 196-08675
Sodium deoxycholate	Wako	Cat#:194-08311
Tris(hydroxymethyl) aminomethane (Tris base)	Nacali tesque	Cat#: 35406-75

**Critical commercial assays**		

QIAquick PCR Purification Kit	QIAGEN	Cat#: 28106
NucleoBond Xtra Midi	TaKaRa	Cat#: 740410
ONE -Glo Ex Luciferase Assay	Promega	Cat#: E8110
ONE -Glo Lysis buffer	Promega	Cat#: E2661
Bio-Rad Protein Assay Dye Reagent Concentrate	Bio-Rad	Cat#: 500-0006
Xfect Transfection Reagent	TaKaRa	Cat#: 631318
Electroporation Cuvettes Green 2mm gap	NEPA Gene	Cat#: EC-002 S

**Experimental models: Cell lines**		

*Drosophila* ovarian somatic cells (OSCs)	Niki et al,^14^ Saito et al.^15^	N/A

**Oligonucleotides**		

Smt3_For (Forward primer) GACGATGACAAGCTTGGTACCATGTCTGACGAAAAGAAGGGAGGT	Thermo Fisher	Wu et al.^1^
Smt3 _Panx4KR-Rev (Reverse primer) CCTCATCGGAGCTTCCATGCTTCCGCCACCAGTCTGCTGCTGGTA	Thermo Fisher	Wu et al.^1^
Smt3_G87A_G88A_Panx4KR-Rev (Reverse primer) CTCTAGCCTCATCGGAGCTTCCATGCTTGCGGCTGCAGTCTGCTGCTGGT	Thermo Fisher	Wu et al.^1^

**Recombinant DNA**		

pAc-Myc-EGFP	Wu et al.^1^	N/A
pAc-Myc-SUMO	Wu et al.^1^	N/A
pAc-λN-HA-3×Flag	Wu et al.^1^	N/A
pAc-λN-HA-3×Flag-Panx	Wu et al.^1^	N/A
pAc-λN-HA-3×Flag-Panx-4KR	Wu et al.^1^	N/A
pAc-λN-HA-3×Flag-SUMO-GG-Panx-4KR	Wu et al.^1^	N/A
pAc-λN-HA-3×Flag-SUMO-AA-Panx-4KR	Wu et al.^1^	N/A
pSUb-14boxB-Luciferase	Wu et al.^1^	N/A

**Software and algorithms**		

SnapGene version 5.1.7	SnapGene	https://www.snapgene.com/

**Other**		

NanoDrop One	Thermo Fisher	Cat#: ND-ONE-W
Bioruptor	Sonic Bio	Cat#: UCD-250
Nucleofector II	Lonza	Cat#: AAB-1001
Cytation5	BioTek	Cat#: CYT5MV

## Materials and equipment

**Table:** TE buffer

Reagent	Final concentration	Amount
Tris-HCl (1 M, pH 7.9)	10 mM	500 μL
EDTA (0.5 M, pH 8.0)	1 mM	100 μL
Milli-Q	N/A	Up to 50 mL
Total	N/A	50 mL

Store for 6 months at R.T.

**Table:** OSCs culture medium

Reagent	Final concentration	Amount
Shield and Sang M3 Insect Medium	N/A	154 mL
Fly extract	10 %	20 mL
Fetal bovine serum	10 %	20 mL
Glutathione (60 mg/mL)	0.6 mg/mL	2 mL
Insulin (1 mg/mL)	10 μg/mL	2 mL
Penicillin-Streptomycin (100×)	1×	2 mL
Total	N/A	200 mL

Store for 1 month at 4°C.

***Note:*** Fly extract should be thawed at 37°C, centrifuged at 10,000 × *g* for 5 min to remove debris, and sterilized using a 0.22-μm filter before use in OSC culture medium.

**Table:** 4x SDS sample buffer

Reagent	Final concentration	Amount
Glycerol	37.5%	7.5 mL
Tris-HCl (0.5 M, pH 6.8)	0.2 M	8 mL
Sodium deoxycholate	4% (W/V)	0.8 g
Bromophenol Blue	0.04% (W/V)	8 mg
Milli-Q	N/A	Up to 20 mL
Total	N/A	20 mL

Store for 6 months at R.T.

**Table:** SDS sample buffer containing 2-Mercaptoethanol

Reagent	Final concentration	Amount
4x SDS sample buffer	1 ×	250 μL
2-Mercaptoethanol	0.1%	1 μL
Milli-Q	N/A	Up to 1 mL
Total	N/A	1 mL

Use immediately after preparation. Do not store unused portions.

**Table:** 0.5 M Phosphate buffer

Reagent	Final concentration	Amount
Na_2_HPO_4_-12H_2_O	0.5 M	89.5 g
H_3_PO_4_	N/A	Adjust pH to 7.2
Milli-Q	N/A	Up to 500 μL
Total	N/A	500 μL

Store for 6 months at R.T.

**Table:** Mannitol buffer

Reagent	Final concentration	Amount
Phosphate buffer (pH7.2)	120 mM	6 mL
Mannitol solution (filtrated)	50 mM	2.5 mL
KCl	5 mM	50 μL
MgCl_2_	15 mM	375 μL
Milli-Q	N/A	16 mL
Total	N/A	25 mL

Syringe filtration by 0.22 μm filter. Store for 6 months at 4°C.

## Step-by-step method details

### Construction of plasmids expressing SUMO-GoI fusion proteins

**Timing: 4 days**

These steps describe the procedures of inserting SUMO-GG or SUMO-AA fragments between λN-HA-3×FLAG and Gene of interest (GoI) to generate plasmids expressing SUMO-GoI fusion proteins (Figure 2A). Although this protocol is broadly applicable to various proteins of interest, Panx was used as an example throughout this protocol for clarity. Details on the cloning of the GoI, including Panx, and the generation of lysine-to-arginine (K→R) mutants are provided in a note at the end of this section.

The experimental timeline was as follows. On Day 1, the PCR-amplified DNA fragment was ligated into the plasmid vector, and the recombinant construct was transformed into Escherichia coli, followed by overnight incubation. On Day 2, individual colonies were picked from ampicillin-containing plates, and the desired plasmid was screened by colony PCR. The selected clones were then submitted for Sanger sequencing to confirm the insert sequence. On Day 3, upon receiving and verifying the sequencing results from the external service provider, *E. coli* harboring the correct plasmid was inoculated into a large volume of LB medium and cultured overnight. On Day 4, plasmid DNA was purified on a large scale for subsequent transfection experiments.

1. Digest 1 μg of a backbone plasmid, pAc-λN-HA-3×Flag-Panx-4KR, using KpnI-HF restriction enzyme with standard protocol.

***Note:*** A unique KpnI site is located between the λN-HA-3×Flag tag and Panx-4KR. The parental pAc-λN-HA-3×Flag vector contains a unique KpnI site in its multiple cloning site (Figure 2B). The GoI must be inserted downstream of the KpnI site to preserve this site for subsequent insertion of the SUMO fragment.

2. Purify the digested plasmid using the QIAquick Spin Kit according to the manufacturer’s instructions.
3. Set up the following PCR on ice to amplify DNA fragments encoding SUMO-GG or SUMO-AA.

**Table:** PCR reaction mix

Reagent	Amount
2×PrimeSTAR Max 2	12.5 μL
pAcM-SUMO (10 ng/μL)	1 μL
Smt3_For (10 μM)	0.5 μL
Smt3 _Panx4KR-Rev (SUMO-GG, 10 μM) or Smt3_G87A_G88A_Panx4KR-Rev (SUMO-AA, 10 μM)	0.5 μL
Milli-Q	To a final volume of 25 μL
Total	25 μL

***Note:*** Use Smt3_Panx4KR-Rev for SUMO-GG and Smt3_G87A_G88A_Panx4KR-Rev for SUMO-AA. The reverse primer sequence should be designed according to each GoI sequence.

**Table:** PCR condition

Steps	Temperature	Time	Cycles
Initial denaturation	98°C	2 min	1
Denaturation	98°C	10 sec	35 cycles
Annealing	60°C	5 sec	
Elongation	68°C	3 sec	
Hold	4°C	–	–

***Note:*** During the design of the reverse primer used in this PCR reaction, a GG→AA mutation is introduced at the C terminus of SUMO to prevent proteolytic cleavage by endogenous SUMO proteases. Simultaneously, a short linker (Ala–Ser or Gly-Ser) is inserted between SUMO and Panx to provide minimal flexibility (Figures 2C and 2D).

4. Analyze the PCR products by agarose gel electrophoresis and confirm that the band sizes are expected.
5. Excise the correct bands from the gel and purify the SUMO-GG or SUMO-AA DNA fragments.
6. Purify the DNA fragments using the QIAquick Spin Kit according to the manufacturer’s instructions.

**CRITICAL:** Ensure complete removal of ethanol during the final wash step. Residual ethanol can significantly reduce NEBuilder HiFi assembly efficiency and result in few or no bacterial colonies.

7. Determine the concentration of vector and insert DNA concentration using NanoDrop one.

**Pause point:** After concentration measurement, purified vector and insert DNA can be stored at −20°C for up to 1 month. For optimal assembly efficiency, avoid repeated freeze–thaw cycles and store DNA in aliquots if necessary.

8. Assemble the vector and insert DNA using the NEBuilder HiFi DNA Assembly reaction with the following components.

**Table:** NEBuilder HiFi DNA Assembly reaction mix

Reagents	Amount (Insert+)	Amount (Insert-)
2× NEBuilder HiFi DNA Assembly	2 μL	2 μL
Linearized pAc-λN-HA-3×Flag–GoI (10 ng/μL)	1 μL	1 μL
Insert DNA (SUMO-GG or AA) (1 ng/μL)	1 μL	0 μL
Milli-Q	0 μL	1 μL

***Note:*** A no-insert control allows estimation of background colonies arising from vector self-ligation and facilitates assessment of assembly efficiency.

9. Incubate the assembly reactions at 50°C for 15 min.
10. Transform the competent *E. Coli* Mach1 cells with the assembled DNA.
  a. Thaw chemically competent *E. coli* Mach1 cells on 25°C immediately.
  b. Mix 2 μL of NEBuilder reaction with 20 μL of Mach1.
  c. Stay on ice 20 min.
  d. Apply heat shock at 37°C for 30 sec.
  e. Store at 25°C 2 min.
  f. Plate transformed Mach1 on LB agar plate containing 100 μg/mL ampicillin.
  g. Incubate plates 16-18 h at 37°C.
  h. Pick colonies from ampicillin-containing plates.
  i. Isolate plasmids using a plasmid isolation kit (e.g., QIAprep Spin Miniprep kit).
  j. Confirm the sequence of insertion by Sanger sequencing or an alternative DNA sequencing method.

***Note:*** Other chemically competent E. coli strains commonly used for cloning (e.g., DH5α or TOP10) can also be used for transformation.

11. Expand verified clones for large-scale plasmid preparation suitable for transfection into OSCs.
12. Purify plasmid DNA using the Xtra Midi Plasmid Purification Kit according to the manufacturer’s protocol.

***Note:*** Kits suitable for purifying plasmid DNA of sufficient quality for transfection into cultured cells are available from various manufacturers, such as Qiagen and Promega.

13. Store the plasmids in TE buffer at 4°C.

***Note:*** Include appropriate control constructs to enable unambiguous interpretation of SUMO-fusion rescue outcomes. At minimum, prepare non-fused wild-type, non-fused SUMOylation-defective K→R mutants, and non-cleavable SUMO(GG→AA)-fusion versions of the same protein (Figure 2A). When feasible, include cleavable SUMO(GG)-fusion constructs as negative controls to assess deSUMOylation and confirm fusion stability.

***Note:*** The coding sequence (CDS) of the gene of interest (GoI) can be obtained from sequence databases such as FlyBase or other appropriate resources and amplified from OSC cDNA using gene-specific primers. The amplified fragment is then cloned into the. EcoRl and Notl site located downstream of the λN-HA-3×Flag tag using standard methods (same protocol as described above in step 6-9). It is recommended to retain one or more restriction enzyme sites (e.g., KpnI) between the tag and the GoI to facilitate subsequent insertion of SUMO (Figure 2B). Loss-of-function mutants can be generated by introducing lysine-to-arginine (K→R) substitutions using site-directed mutagenesis with mutation-containing primers at selected residues based on reported SUMOylation sites or predicted consensus motifs. Mutations should be confirmed by DNA sequencing, and functional validation is recommended using the tethering assay described below.

### Expression and validation of a SUMO-fusion GoI loss-of-function mutant in cultured cells

**Timing: 2–3 days**

These steps describe the procedures of expressing SUMO-fusion GoI-loss of function mutant in OSCs and for verifying fusion integrity and stability prior to functional rescue assays.

14. Seed 1×10^6^ OSCs in 35-mm dish and culture for 16–20 h at 26°C.
15. Confirm that the cells have reached 60% confluency at the time of transfection.
16. Mix the following reagents in separate tubes.

**Table:** 

<Tube 1>	
Xfect reaction buffer	75 μL
Vector plasmid (1 μg/μL)	3 μL
<Tube 2>	
Xfect reaction buffer	75 μL
Xfect polymer	0.9 μL

***Note:*** Vector plasmid refers to expression constructs encoding GoI, including control and SUMO-fusion variants (e.g., pAc-Myc-EGFP, pAc-λN-HA-3×Flag-GoI, GoI loss-of-function mutant, pAc-λN-HA-3×Flag-SUMO-GG-GoI, and pAc-λN-HA-3×Flag-SUMO-AA-GoI). In this protocol, Panx is used as an example GoI.

17. Prepare transfection complexes according to the manufacturer’s protocol for Xfect (Takara).
18. Aspirate the OSC culture medium and replace it with 2 mL of Shields and Sang M3 Insect Medium, to remove serum from the cell culture.
19. Add the entire nanoparticle complex solution dropwise to the cell culture medium. Rock the plate gently back and forth to mix. Incubate OSCs at 26°C for 3 h.
20. Aspirate the nanoparticle-containing medium and replace it with 2 mL of fresh complete OSC culture medium.
21. Incubate OSCs at 26°C for an additional 21 h.

***Note:*** Here, transient transfection is performed using Xfect (Takara), which is highly recommended due to its high transfection efficiency in OSCs. Alternative methods, such as electroporation-based delivery systems or other lipid-based transfection reagents, may also be applicable depending on the experimental setup. However, transfection efficiency and cell viability should be empirically optimized for each method.

22. Wash the cell layer once with PBS.
23. Harvest cells using Trypsin-EDTA and determine the cell number.
24. Lyse 2×10^6^ OSCs in 100 μL of SDS sample buffer containing 0.1% 2-Mercaptoethanol.
25. Sonicate samples for 5min using a Bioruptor.

**Pause point:** Samples in SDS sample buffer containing 0.1% 2-mercaptoethanol can be stored at −20°C for up to 1 month before boiling and SDS–PAGE analysis. Avoid repeated freeze–thaw cycles.

**Figure 3:**
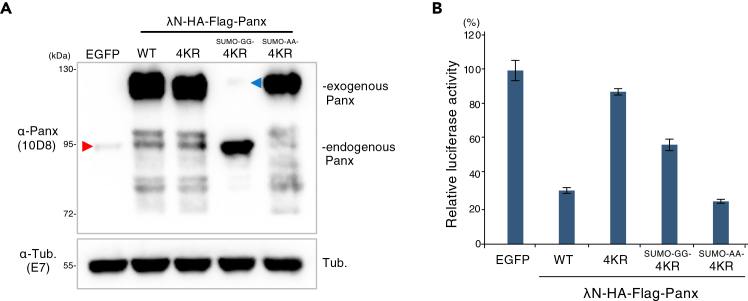
SUMO fusion rescues the transcriptional silencing activity of the Panx 4KR mutant (A) SUMO-GG-Panx 4KR is targeted by endogenous deSUMOylating enzymes. Western blotting with an anti-Panx antibody (10D8) shows that replacement of the C-terminal diglycine motif with alanine residues (GG→AA) enhances the stability of the SUMO-Panx-4KR conjugate. A trace amount of SUMO-GGG-Panx-4KR escapes cleavage by deSUMOylating enzymes (blue arrowhead), whereas the majority migrates to the same position as endogenous Panx (red arrowhead). (B) Forced tethering of WT Panx and Panx mutants. In-frame conjugation of SUMO to the Panx-4KR mutant restores its transcriptional silencing activity. Although λN-fused SUMO-GG-Panx-4KR is largely cleaved, it still shows a weak but detectable restoration of silencing activity. Error bars indicate SD (n = 3).

***Note:*** The GG peptide at the C-terminus of the SUMO protein serves as a target for de-SUMOylation enzymes, which cleave the fusion protein between λN-HA-Flag-SUMO and PoI. In this study, such cleavage was observed for the Panx-4KR construct, even in the case of an in-frame fusion. Consequently, the expression of λN-HA-Flag-SUMO-GG-Panx-4KR resulted in a protein migrating at the position of endogenous Panx due to this cleavage, and a loss of SUMO. Therefore, we replaced the GG-linker with an AA-linker to enhance the stability of the SUMO conjugation to Panx-4KR (Figure 3A).

26. Boil samples at 95°C for 5 min prior to SDS–PAGE.
27. Separate proteins by 7.5% SDS–PAGE.
28. Transfer proteins to membranes for immunoblotting.
29. Probe membranes with antibodies against Protein of interest (PoI) or Tubulin for 1 h.
30. Incubate with HRP-conjugated sheep anti-mouse IgG secondary antibody for 30 min.
31. Detect signals using ECL Prime Western Blotting Detection Reagents.
32. Confirm the expression and the expected molecular weight of SUMO–GoI fusion proteins (Figure 3A).

### Tethering assay based on transient co-transfection of OSCs

**Timing: 2 days**

These steps describe the use of a tethering assay to evaluate the transcriptional repression activity of PoI. In case of Panx, the wild-type protein exhibits repressive activity, whereas substitution of four lysine residues with arginine (4KR) abolishes this repressive activity. Notably, in-frame fusion of SUMO restores the repressive function (Figure 3B).

33. Collect 3 × 10^6^ OSCs per sample into a tube.
34. Wash the cells once with PBS.
35. Resuspend the cells in 100 μL of Mannitol transfection buffer.
36. Add the following plasmids to the cell suspension:

**Table:** 

pUb-14boxB-Luc (300 ng/μL)	2 μL
Vector plasmid (1 μg/μL)	3 μL

***Note:*** Vector plasmids means either constructs expressing (i) the wild-type PoI, (ii) a loss-of-function mutant (e.g., lysine-to-arginine substitutions), or (iii) SUMO-fusion variants (e.g., SUMO-GG or SUMO-AA fused to the mutant PoI) for comparative analysis. Here, Panx constructs (pAc-Myc-EGFP, pAc-λN-HA-3×Flag-Panx, pAc-λN-HA-3×Flag-Panx-4KR, pAc-λN-HA-3×Flag-SUMO-GG-Panx-4KR, or pAc-λN-HA-3×Flag-SUMO-AA-Panx-4KR) are used as representative examples.

37. Transfer each 105 μL sample to an electroporation cuvette.
38. Electroporate the cells using program T-029 on the Nucleofector II Device.

***Note:*** In addition to the cuvette-based Nucleofector II system used in this protocol, other electroporation platforms, including multiwell electroporation systems, can also be used for transient transfection of OSCs after optimization of the program and buffer conditions. Lipid-based transfection reagents, such as Xfect (Takara), are also applicable, although their efficiency should be empirically optimized depending on the experimental conditions.

39. Plate the cells into three wells of a 24-well plate containing 500 μL of culture medium per well.
40. Incubate the cells at 26°C for 24 h.
41. Harvest the cells for the luciferase assay.
  a. Aspirate the culture medium from the 24-well plates.
  b. Wash each well once with PBS.
  c. Add 150 μL Glo Lysis Buffer to each well.
  d. Lyse the cells by shaking at 120 rpm for 10 min at 25°C.
  e. Transfer the cell lysates to 1.5-mL microcentrifuge tube.
  f. Centrifuge the lysates at 15,000 × *g* for 5 min at 4°C.
  g. Collect the cleared supernatant.

42. Measure the luciferase activity.
  a. Dispense 15 μL of ONE-Glo luciferase assay substrate into each well of a round-bottom, white 96-well plate.
  b. Add 15 μL of cleared cell lysate to the substrate and mix gently.
    **CRITICAL:** After centrifugation, carefully collect the supernatant without disturbing the pellet. Avoid aspirating cellular debris, as pellet contamination can increase variability in luciferase and protein measurements.
  c. Measure firefly luciferase activity using a Cytation 5 multi-mode plate reader (BioTek) in luminescence mode (endpoint measurement, 3.0 s integration time, 100 ms delay, top reading, gain 135) without excitation or emission filters (full-spectrum detection, 300–700 nm detection range), using a white opaque 96-well plate. Firefly luciferase emits with a peak around 550–570 nm when using the One-Glo assay.

43. Measure total protein concentration using the Bradford assay.
  a. Mix 5 μL of cleared cell lysate with 500 μL of Bradford reagent.
  b. Transfer 150 μL of the mixture to a flat-bottom, clear 96-well plate.
  c. Measure absorbance at 595 nm using a microplate reader.
44. Normalize luciferase activity to total protein concentration for each sample (Figure 3B).

## Expected outcomes

This protocol is illustrated using Panx as a representative example. It is applicable to functional analyses of proteins that undergo SUMOylation, for which the SUMO acceptor lysine residues have been identified and lysine-to-arginine substitutions at these sites result in a clear loss-of-function phenotype. In such cases, loss-of-function analyses have already established that disruption of the modification correlates with impaired protein activity. Under these conditions, the present protocol enables a gain-of-function approach to directly evaluate whether SUMO conjugation itself is functionally required. By restoring SUMO attachment through a stable SUMO fusion, this approach allows causal assessment of the contribution of SUMOylation to protein function beyond conventional mutagenesis-based analyses.

Western blot analysis demonstrated that in-frame fusion of SUMO to the N terminus of the KR mutant requires modification of the SUMO C-terminal motif to ensure fusion stability. SUMO–Panx fusion proteins retaining the native C-terminal diglycine (GG) motif were efficiently cleaved between SUMO and Panx, consistent with processing by endogenous deSUMOylating enzymes. As a result, these constructs predominantly migrated at a molecular weight corresponding to non-fused Panx. In contrast, substitution of the diglycine motif with alanine residues (GG→AA) effectively blocked proteolytic cleavage, yielding stable SUMO–Panx fusion proteins that migrated at the expected higher molecular weight (Figure 3A).

In the λN–boxB tethering assay, wild-type Panx robustly repressed reporter gene expression, whereas the SUMOylation-defective Panx 4KR mutant exhibited a pronounced loss of transcriptional repression activity. Notably, in-frame fusion of a non-cleavable SUMO variant (SUMO-AA) to the N terminus of the Panx 4KR mutant restored repression activity to levels comparable to those of wild-type Panx (Figure 3B). In contrast, fusion with a cleavable SUMO retaining the C-terminal GG motif (SUMO-GG) resulted in only limited restoration of repression. Together, these results demonstrate that stable SUMO attachment is sufficient to restore Panx-mediated transcriptional repression, supporting a direct and functional requirement for SUMOylation in Panx activity.

## Limitations

In this protocol, an N-terminal SUMO fusion strategy was employed because the lysine residues predicted to undergo SUMOylation in Panx are located near the N terminus. However, the applicability of this approach may depend on the positional context of SUMO acceptor lysines within the target protein. When functionally critical SUMOylation sites are buried within the protein core or located deep within specific domains, fusion of SUMO to the N or C terminus may not adequately recapitulate the native modification state and may therefore fail to restore protein function.

Consistent with this limitation, we previously observed that N-terminal SUMO fusion did not restore the activity of Setx, whereas fusion at the C terminus resulted in partial functional rescue.^1^ These observations suggest that the spatial positioning of the fused SUMO moiety relative to the target protein is a key determinant of functional outcome. Accordingly, the length, flexibility, and composition of the linker connecting SUMO to the target protein are likely to be critical parameters that require empirical optimization. Alternative linker designs may alter the distance and orientation between SUMO and the target protein and could therefore lead to different degrees of functional rescue. Systematic testing of linker length and flexibility may therefore be necessary when extending this approach to other proteins.

Furthermore, the non-cleavable SUMO fusion used in this protocol (GG→AA) is resistant to processing by endogenous deSUMOylating enzymes. While this design ensures stable SUMO attachment, it precludes analysis of the dynamic and reversible cycles of SUMO conjugation and deconjugation that occur under physiological conditions. Consequently, this experimental system may not be suitable for investigating regulatory processes that depend on reversible SUMOylation.

Finally, in mammals, multiple SUMO isoforms (SUMO1 and SUMO2/3) exist and can exert distinct biological functions.^2^ Fusion of different SUMO isoforms to a target protein may allow dissection of isoform-specific roles. Moreover, the contribution of branched poly-SUMO chains could potentially be evaluated by fusing SUMO mutants in which lysine residues are substituted with arginine (SUMO KR variants), thereby preventing poly-SUMO chain formation.^2^ However, these extensions were not explored in the present protocol and represent important directions for future methodological development.

## Troubleshooting

### Problem 1

High background colony formation is observed in the no-insert control, and/or the number of colonies obtained from insert-containing assemblies is low (Step 10).

### Possible cause

• Inefficient NEBuilder HiFi DNA Assembly, resulting in poor incorporation of the insert (insert+).
• Incomplete restriction digestion of the vector backbone, leading to residual intact plasmid and background colony formation in the no-insert control (insert-).

### Potential solution

• Optimize the vector-to-insert molar ratio prior to assembly (Step 8).
• Ensure complete removal of residual ethanol after DNA purification, as ethanol carryover can reduce the efficiency of NEBuilder assembly (Step 6).
• Extend the NEBuilder assembly incubation time to 1 h to improve assembly efficiency (Step 9).
• Redesign primers to increase the length of homologous overlaps between the vector backbone and insert DNA, thereby ensuring sufficient sequence homology for efficient assembly.
• Increase digestion time by restriction enzyme to minimize residual undigested vector that can give rise to background colonies (Step 1).
• Verify the activity and freshness of the selection antibiotic in LB agar plates to ensure effective selection (Step 10).

### Problem 2

No detectable signal of the SUMO-GoI fusion protein is observed in Western blotting (Step 32).

### Possible cause

• Low expression level of the SUMO-GoI fusion protein.
• Inefficient gene delivery.
• Inappropriate detection using anti-Flag or anti-HA antibodies in Western blotting

### Potential solution

• Optimize Xfect-mediated transfection conditions, including cell density, DNA amount, reagent ratio, and medium replacement timing, to improve expression of the SUMO-GoI fusion protein.
• Increase the amount of total protein loaded per lane for SDS-PAGE (Step 27).
• Perform Western blotting using an antibody against PoI (Step 29). In OSCs, λN-HA-3×Flag-SUMO-GG-Panx-4KR is cleaved by endogenous deSUMOylation enzymes, resulting in separation into λN-HA-3×Flag-SUMO (∼16 kDa) and Panx-4KR (∼90 kDa). The HA- and Flag-tagged SUMO fragment is too small to be resolved on 7.5% SDS-PAGE, which can compromise detection with anti-HA or Flag antibodies.

### Problem 3

Large variability is observed in luciferase activity and/or total protein measurements between replicates (Steps 42 and 43).

### Possible cause

• Rapid aspiration of culture medium from 24-well plate (Step 41-a).
• Disturbance of cleared lysates after centrifugation, resulting in inconsistent sampling (Step 41-g).
• Inaccurate pipetting of small volumes, particularly during Bradford assays using low lysate input (Step 43-a).
• Insufficient mixing of lysates with Bradford reagent (Step 43-a).

### Potential solution

• Carefully aspirate the culture medium manually using a pipette rather than an automatic suction device (Step 41-a). Because electroporation causes substantial cellular damage and generates abundant debris, rapid removal of the medium using an automatic aspirator can detach cells that have adhered to the bottom of the culture well.
• After centrifugation, handle lysates gently and avoid disturbing the pellet; collect samples from the upper portion of the supernatant (Step 41-g).
• When transferring small volumes (e.g., 5 μL for Bradford assays), minimize contact between the liquid and the outer surface of the pipette tip to improve volume accuracy (Step 43-a).
• Mix lysates thoroughly with Bradford reagent by sealing the tube and shaking manually; vortexing alone may be insufficient to achieve homogeneous mixing (Step 43-a).

### Problem 4

Fusion of SUMO fails to restore the activity of the target protein (Step 44).

***Note:*** Despite SUMO fusion, the functional activity of the target protein is not recovered. This problem is described in a general context and is not limited to Panx, but rather applies broadly to other proteins of interest investigated by individual researchers.

### Possible cause

• Loss of the SUMO-target protein linkage.
• The position of SUMO fusion is not appropriate.
• SUMOylation does not regulate the target protein.

### Potential solution

• The C terminus of the fused SUMO retains the GG motif, allowing cleavage by endogenous deSUMOylating enzymes. GG motif should be replaced to AA to prevent digestion by deSUMOylating enzymes.
• Test SUMO fusion not only at the N terminus but also at the C terminus of the target protein. In these constructs, replace the C-terminal GG motif of SUMO with AA to prevent re-conjugation by endogenous SUMO enzymes, which could otherwise result in conjugation to lysine residues on other proteins.
• Reconsider the underlying hypothesis that “SUMOylation regulates the target protein.” However, as discussed in the Limitations section, successful functional rescue by in-frame SUMO fusion critically depends on the compatibility between SUMO and the target protein. Because this experimental system has multiple technical and conceptual constraints, the absence of functional rescue alone is insufficient to definitively refute the hypothesis.

## Resource availability

### Lead contact

Further information and requests for resources and reagents should be directed to and will be fulfilled by the lead contact, Kensaku Murano (kmurano@keio.jp).

### Technical contact

Technical questions on executing this protocol should be directed to and will be answered by the technical contacts, Rin Imai (rinimai29525@keio.jp), Yaning Wu (yaningwu@keio.jp) and Kensaku Murano (kmurano@keio.jp).

### Materials availability

Plasmids used in this study are listed in the key resources table and are available from the lead contact upon request.

### Data and code availability

• This paper does not report original code.
• Any additional information required to reanalyze the data reported in this paper is available from the lead contact upon request.

## Acknowledgments

This work was supported by funding from 10.13039/501100001691JSPS 10.13039/501100001691KAKENHI grant nos. 17K08644, 20H03439, 23K27370, and 24K22039, and from a 10.13039/100008608Sumitomo Foundation Research Grant 200672 to K.M.

## Author contributions

K.M. and Y.W. conceived the protocol. R.I., K.M., and R.K. wrote the manuscript. K.M. and Y.W. produced the plasmids used in this paper. Transfection, Immunoblotting, and reporter assay were performed by R.I., R.K., and K.M.

## Declaration of interests

The authors declare no competing interests.

## Declaration of generative AI and AI-assisted technologies in the writing process

During the preparation of this work, the authors used ChatGPT, Grammarly, and DeepL to improve the readability and language of the manuscript. After using these tools, the authors reviewed and edited the content as needed and take full responsibility for the content of the published article.
